# Lipid mediators in plasma of autism spectrum disorders

**DOI:** 10.1186/1476-511X-11-160

**Published:** 2012-11-21

**Authors:** Afaf El-Ansary, Laila Al-Ayadhi

**Affiliations:** 1 Biochemistry Department, Science College, King Saud University, Riyadh, Saudi Arabia; 2 Department of Physiology, Faculty of Medicine, King Saud University, Riyadh, Saudi Arabia; 3 Autism Research and Treatment Center, Riyadh, Saudi Arabia; 4 Shaik AL-Amodi Autism Research Chair, King Saud University, Riyadh, Saudi Arabia

**Keywords:** Autism, Inflammation, Prostaglandins, Leukotrienes, Isoprostane, Arachidonic acid

## Abstract

**Background:**

Inflammation is increasingly recognized as being of both physiological and pathological importance in the immature brain. Cerebellar pathology occurs in autism, as a neurodevelopmental disorder with genetic and environmental origins. The genesis of this disorder is still not understood but inflammation in utero or early in childhood is an environmental risk factor.

**Methods:**

Prostaglandin E2 (PGE2), cysteinyl leukotriene as two important lipid mediators together with 8 isoprostane as marker of oxidative stress were measured using ELISA in plasma of 20 male autistic patients compared to 19 age and gender matching control participants.

**Results:**

PGE2, leukotrienes and isoprostanes recorded significantly elevated levels in autistics compared to controls. Role of these measured parameters in inflammation and autoimmunity as two etiological factors in autism were discussed in details.

**Conclusion:**

Receiver Operating Characteristic (ROC) curve analysis shows satisfactory values of area under the curve (AUC) which could reflect the high degree of specificity and sensitivity of the altered PGE2, leukotrienes and isoprostanes as predictive biomarkers in autistic patients from Saudi Arabia.

## Background

Autism spectrum disorders (ASDs) are currently one of the leading causes of developmental disability with approximately 1% children affected [1]. Etiologically, many different factors are involved. One of the most striking features of autism and other developmental disorders is that cognitive, social and sensory/motor development usually progress symptom free for several months to years but then is followed by a period of retardation where some skills fail to develop or do so but behind schedule, a period of regression where some acquired skills are lost, or a period of intrusion where acquired skills are overshadowed by the appearance of behaviors aberrant in form or frequency [2,3]. Importantly, the period when symptoms first begin to appear may represent the time when environmental toxicants have accumulated in the brain to critical levels and/or the deleterious effects of earlier exposure may become manifest through perturbation of normal ontogenic development of brain pathways [4]. Furthermore, it is thought that certain individuals may be more sensitive to toxicants because of a genetic predisposition. Exposure during critical periods may disrupt neurobehavioral development by altering neural migration, circuitry, and/or synaptogenesis of brain areas required for expression of these behaviors [5]. A dysregulated immune response, accompanied by enhanced oxidative stress, abnormal mitochondrial metabolism, and impaired lipid metabolism seemingly represents the common molecular underpinning of certain neurodevelopmental disorders among which is autism spectrum disorders (ASD). Understanding and confirming the role of these pathways in the aetiology of autism are important for the definition of pharmacological therapies able to ameliorate clinical symptoms [6-8]. Most recently, the strongest evidence was given for immune dysregulation/inflammation and oxidative stress, followed by toxicant exposures and mitochondrial dysfunction as trends in physiological abnormalities in autism spectrum disorders [9].

Arachidonic acid (AA) is metabolized by means of two pathways: cyclooxygenase, leading to the formation of prostaglandins, and lipoxygenase, leading to the production of leukotrienes. Their concentrations are reportedly increased in brain injury [10] and is directly related to the increase of blood–brain barrier (BBB) permeability [11].

Prostaglandin E2 (PGE2) is a compound derived from membrane phospholipids and an important mediator of synaptic plasticity, pain response, sleep/ awake cycle and is believed to be associated with inflammation in the brain [12-15]. Both stimulant and depressant effects of PGs on the CNS have been reported following their injection into the cerebral ventricle and the firing rates of individual brain cells may be increased or decreased after iontrophoric applications of PGs [16]. PGs have been proposed to modulate catecholaminergic [17], serotoninergic [18] and cholinergic [19] neurons in the CNS. There is also accumulating data suggesting possible modulatory role of PGs on dopamine mediated behavior [20].

Several in vitro markers of oxidative stress are available, but most are of limited value in vivo because they lack sensitivity and/or specificity or require invasive methods [16]. Isoprostanes are prostaglandin (PG) –like substances that are produced in vivo independently of cyclooxygenase (COX) enzymes, primarily by free radical- induced peroxidation of arachidonic acid [21]. Oxidation of docosahexaenoic acid, an abundant unsaturated fatty acid in the central nervous system, results in the formation of isoprostane-like compounds, termed neuroprostanes [22].

Convincing experimental data indicate that PGs function in mostly pathological processes in the CNS, including, fever induction, learning and memory, and excitotoxic brain injury such as stroke, epilepsy [23]. Tamiji and Crawford [24] recorded deficits associated with the release of arachidonic acid from membrane phospholipids and its subsequent metabolism to PGs in ASD. This was supported by a more recent study in which impaired fatty acids profile and decreased concentrations of Phosphatidylserine (PS), Phosphatidylcholine (PC),and Phosphatidylethanolamine (PE) were recorded in autistic patients compared to healthy controls [25]. This information motivate our interest to measure PGE2, cysteinyl leukotriene and 8-isoprostane in plasma of autistic patients in a trial to investigate the role of these lipid mediators related to AA and Phospholipids (PS, PC and PE) in the pathology of autism.

## Methods

### Reagents and chemicals

Ethyl acetate and potassium hydroxide used for extraction of 8 isoprostane were of analytical grade and obtained from Sigma-Aldrich (Taufkirchen, Germany).

### Participants and methods

The study protocol followed the ethical guidelines of the most recent Declaration of Helsinki (Edinburgh, 2000). All subjects enrolled in the study (20 autistic males and 19 control males) had written informed consent provided by their parents and assented to participate if developmentally able. They were enrolled through the ART Center (Autism Research & Treatment Center) clinic. The ART Center clinic sample population consisted of children diagnosed on the autism spectrum disorder (ASD). The diagnosis of ASD was confirmed in all subjects using the Autism Diagnostic Interview-Revised (ADI-R) and the Autism Diagnostic Observation Schedule (ADOS) and 3DI (Developmental, dimensional diagnostic interview). The ages of all autistic children participated in the study were between 4–12 years old. All were simplex cases. All are negative for fragile x gene study. The control group recruited from well baby clinic at king Khaled university hospital with mean age 4–11 years old. Subjects were excluded from the investigation if they had dysmorphic features, or diagnosis of fragile X or other serious neurological (e.g., seizures), psychiatric (e.g., bipolar disorder) or known medical conditions. All participants were screened via parental interview for current and past physical illness. Children with known endocrine, cardiovascular, pulmonary, liver, kidney or other medical disease were excluded from the study.

### Samples collection

Blood samples were collected in the morning following at least 10 hour period of fasting. Plasma was collected using standard clinical practices and stored at −80°C until thawed for analysis.

#### Assay of 8-Isoprostane

8 isoprostane was extracted and saponified from plasma of autistic and control participants using ethyl acetate and potassium hydroxide (KOH). The aqueous solution was diluted using dilution buffer and 8 isoprostane then was measured using ELISA kit, product of Detroit R and D, Inc according to the instructions of the manufacturer. The plate was read at 450 nm together with serial concentrations of standard.

#### Assay of PGE2

PGE2 was measured using a diagnostic kit, a product of Uscn, Life Science Inc, USA. This assay employs the competitive inhibition enzyme immunoassay technique in which a microplate was pre-coated with monoclonal antibody specific for human PGE2. A competitive inhibition reaction is launched between biotin labeled human PGE2 and unlabeled human PGE2 (Standards or samples) with the pre-coated antibody specific for human PGE2. After incubation the unbound conjugate is washed off. After washing, avidin conjugated to Horseradish peroxidase (HRP) is added to each microplate well and incubated. The amount of bound HRP conjugate is reverse proportional to the concentration of PGE2 in the sample. After addition of the substrate solution, the intensity of colour developed is reverse proportional to the concentration of PGE2 in the sample. The minimum detectable concentration of PGE2 is typically less than 10.79 pg/ml.

#### Assay of cysteinyl leukotriene

Cysteinyl leukotriene was measured in plasma of autistic patients using a non-radioactive, safe ELISA kit, a product of Enzo life Science. The coated well immuno-enzymatic assay for the quantitative measurement of LTC4 /D4 /E4 utilizes a multi-clonal anti-LT antibody and a LT- HRP conjugate. The intensity of colour is measured spectrophotometrically at 405 nm in a microplate reader and it is inversely proportional to the LT concentration. The detection limit range is 78.1-2500 pg/ml and sensitivity in this assay is 26.6 pg/ml.

#### Statistical analysis

An SPSS computer program was used. Results were expressed as mean ± S.D. and all statistical comparisons were made by means of independent t-Test with P ≤ 0.005 was considered significant. Receiver Operating Characteristics analysis (ROC) was performed. Area under the curve, cut off values together with degree of specificity and sensitivity were calculated. ROC curves are constructed by plotting the false positive rate (i.e. 100-specificity) against the true positive rate (i.e. sensitivity).

These have been widely accepted as standard tools for evaluating the performance of diagnostic tests. The AUC is an overall summary of diagnostic accuracy, incorporating both components of accuracy, i.e., sensitivity and specificity, into a single measure. The AUC has been widely used as a quantitative index of the performance of a biomarker in a variety of applied fields; it is a simple and convenient overall measure of diagnostic test [26].

## Results

**Figure 1:**
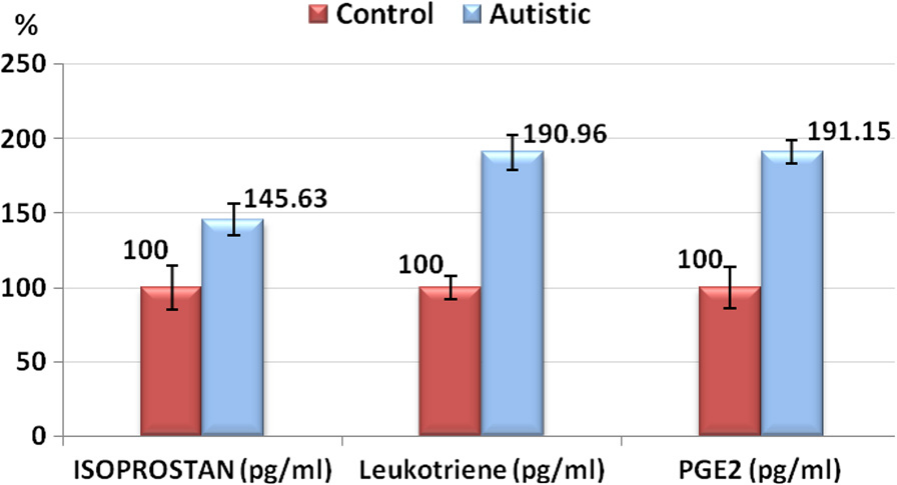
Percentage change with error bars of isoprostan (pg/ml), leukotriene (pg/ml) and PGE2 (pg/ml) in autistic group compared to control.

Results are presented as Mean ± S.D. of 8- isoprostane, leukotriene and PGE2 of 20 autistic patients and 19 age and gender-matching controls. It could be easily noticed that the three measured parameters were significantly higher in autistic patients, recording an increase of 45.65, 90.9 and 91.15% for 8- isoprostane, leukotriene and PGE2 respectively (Table 1 and Figure 1).

**Table 1:** Levels of 8-isoprostane, leukotrienes and PGE2 in plasma of autistic patients compared to healthy controls

**Parameters**	**Groups**	**N**	**Mean ± S.D.**	**P value**
Isoprostanes (pg/ml)	Control	19	68.27 ± 10.00	0.001
	Autistic	20	99.42 ± 10.76	
Leukotriene (pg/ml)	Control	19	190.42 ± 15.27	0.001
	Autistic	20	363.62 ± 43.12	
PGE2 (pg/ml)	Control	19	217.97 ± 30.00	0.001
	Autistic	20	416.64 ± 31.52	

**Figure 2:**
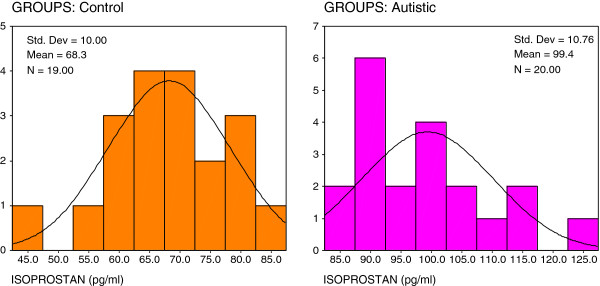
Normal distribution for control and autistic groups in isoprostan (pg/ml).

**Figure 3:**
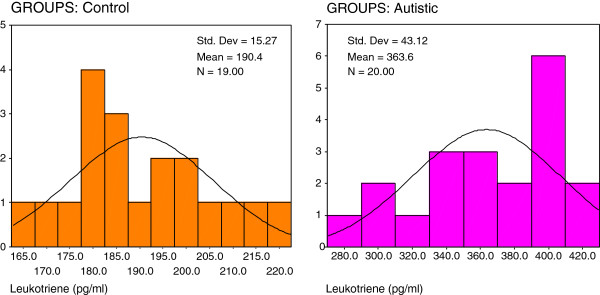
Normal distribution for control and autistic groups in Leukotriene (pg/ml).

**Figure 4:**
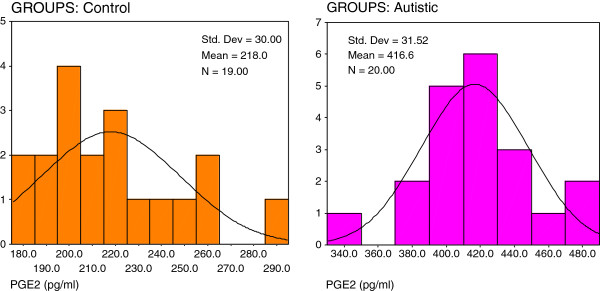
Normal distribution for control and autistic groups in PGE2 (pg/ml).

Figures (2, 3, 4) present the normal distribution of the measured parameters. Figure 2 demonstrates that 12/20 of autistic patients recorded isoprostane levels higher than 100 pg/ml, while 8/20 recorded values greater than 85 pg/ml as the maximum concentration in control subjects. 18/20 of control subjects, recorded isoprostane levels lower than 85 pg/ml. Figure 3 shows that 20/20 of autistic patients recorded leukotriene levels higher than 280 pg/ml compared to a remarkable lower value less than 200 pg/ml in 15/19 of control subjects. Figure 4 shows that lower level of PGE2 in autistic patients (360 pg/ml) was much greater than the highest concentration in control (280 pg/ml).

**Figure 5:**
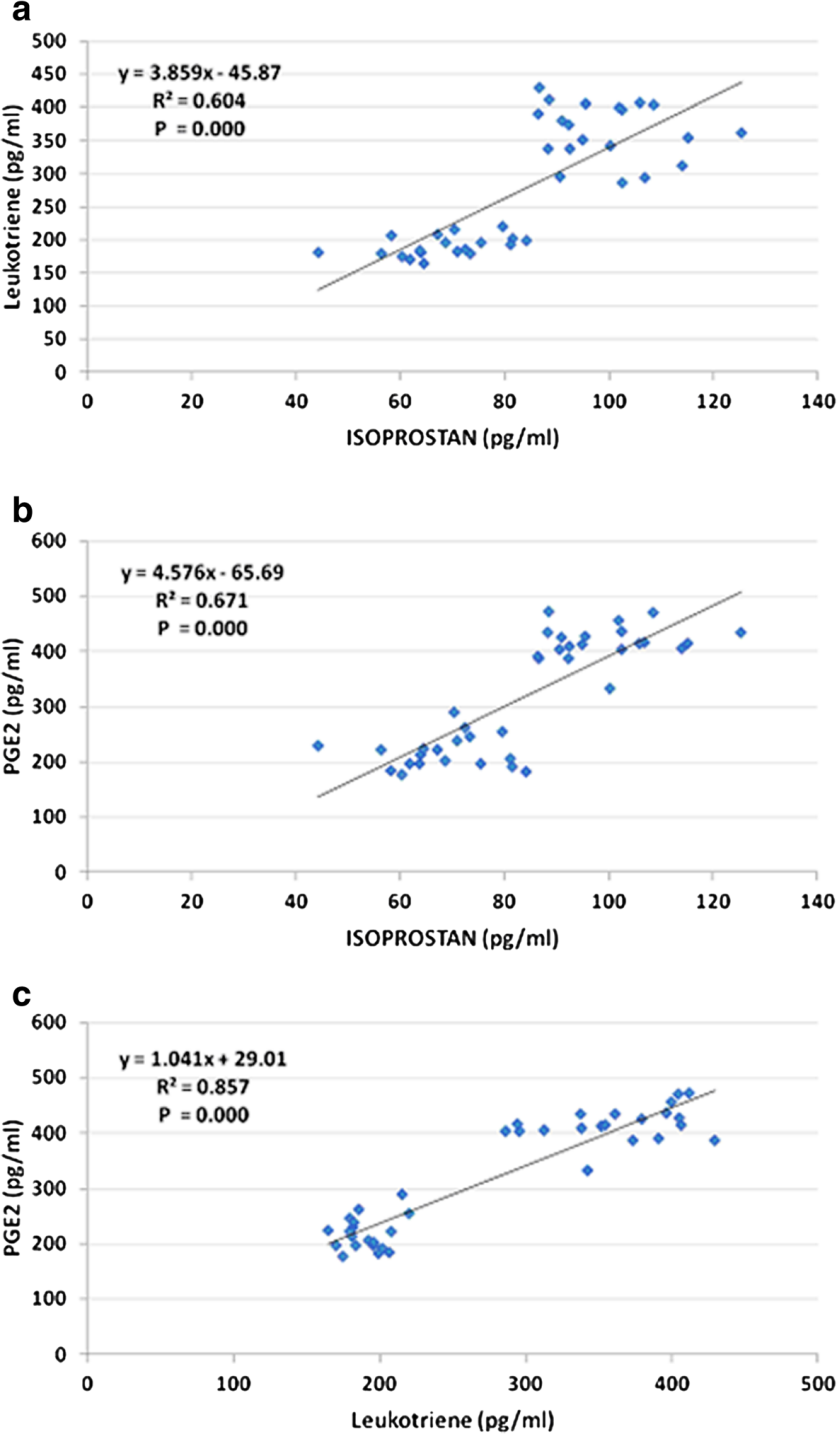
Pearson correlations between the three measured parameters with the best fit line.

**Figure 6:**
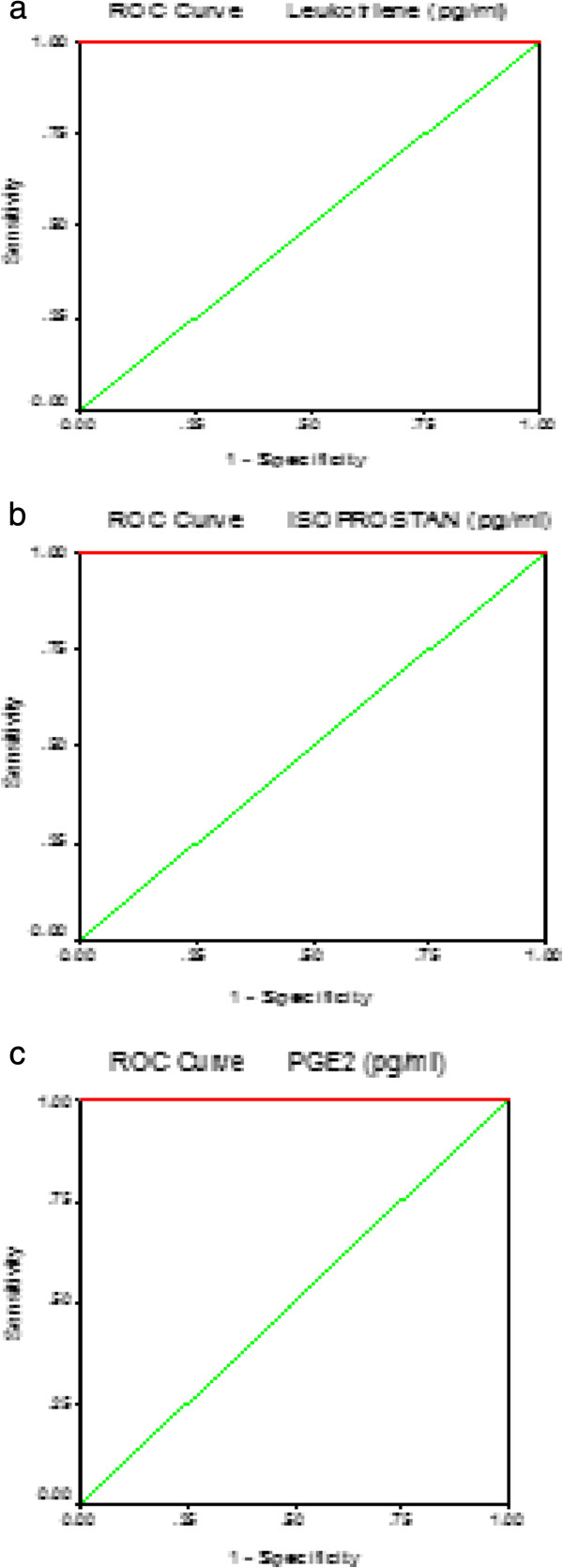
ROC Curve of isoprostan (a)leukotriene (b) and PGE2(c) in autistic group.

Table 2 and Figure 5 demonstrate the Pearson correlations between the three measured parameters. It could be easily seen that 8-isoprostane, leukotrienes and PGE2 are positively correlated with P values < 0.001. Table 3 together with Figure 6 show the ROC analysis of the measured parameters. Area under the curve, specificity and sensitivity together with the best cut off values.

**Table 2:** By using statistical analysis program (SPSS) which includes the pearson correlation test, the correlation was done between all parameters; the results showed that there was a significant correlation difference between the following parameters

**Parameters**	**R (Pearson Correlation)**	**Sig.**	
Isoprostan ~ Leukotriene	0.777	0.001	P^a^
Isoprostan ~ PGE2	0.820	0.001	P^a^
Leukotriene ~ PGE2	0.926	0.001	P^a^

^**a**^ Positive Correlation.

**Table 3:** ROC analysis of the three measured parameters

**Parameter**	**Area under the curve**	**Cutoff value**	**Sensitivity %**	**Specificity %**
Isoprostan	1.000	78.270	100.0%	78.9%
Leukotriene	1.000	205.689	100.0%	84.2%
PGE2	1.000	247.968	100.0%	84.2%

## Discussion

The demonstration of oxidation of membrane phospholipids (PL) led to a new fruitful era with a continuous flow of considerable research devoted to chemistry, biochemistry and medicine [27]. Hydroperoxides derived from AA serve as modulators of the enzymes involved in the biosynthesis of prostaglandins and leukotrienes [28-30]. It is well known that non-enzymatic free radical-induced cyclooxygenation of AA and its phospholipid esters through the isoprostane pathway gives rise to four structurally isomeric families of phospholipid endoperoxide stereoisomers of PGs which were coined isoprostanes (isoPs) [21,27]. As electrophils, isoprostanes have exceptional reactivity to biological nucleophiles such as proteins and DNA [31].

In the present study, the remarkable increase of isoprostanes in plasma of Saudi autistic patients compared to healthy controls (Table 1), could be easily related to the oxidative stress as an accepted mechanism in the etiology of autism. This suggestion could be supported through considering the previous work of Ming et al. [32] in which they recorded urinary excretion of 8-isoprostane (8-iso-PGF2a) a class of autoxidation products generated from AA acid by a free radical initiated process, in children with autism compared to age-matched controls. Both finding are consistent with the recent work of El-Ansary et al. [33] in which they proved that autistic patients from Saudi Arabia have significantly lower level of AA and suggested impaired fatty acid profile as a diagnostic marker of autism. Elevated plasma and urinary isoprostans together with the diminished level of AA could be related to the impaired inflammatory responses seen in autistic patients [34].

The contribution of isoprostane- related oxidative injury to autism was explored by autoimmunity as an etiological factor of autism [35]. Autoimmunity, an abnormal immune reaction in which the immune system becomes primed to react against body organs. It can result from an immune response against altered self proteins, e.g., modified by adduction of lipid-derived electrophiles generated by oxidative injury. IgG, IgA ,IgM and myelin basic protein anti-brain auto antibodies were present in high percentage of sera from children with autism compared to healthy children [36]. Nine neuron-specific antigens, three encephalitogenic and microfilaments were among the autoimmune-attacked proteins. The possible operation of an immune response against altered self proteins in autism was demonstrated by Lu [35]. The occurrence of 2-pentylpyrrole, iso [3] LGE2-protein adducts and carboxyethylpyrrole (CEP) protein modifications in brain and blood from autistic individuals was proved [37,38].

Moreover, immunohistochemical analysis of brain tissues such as cerebellum, hippocampus and neocortical regions has shown significant CEP-staining and iso [3]LGE2-protein adduct in the white matter in every autistic cases compared to control age-matched cases which suggest elevated oxidative damage in these brain regions in autism [27].

It has been recognized for many years that leukotrienes play an important role in mediating various effects of the allergic reaction. Recent evidence has shown that they play a role in many diseases. Leukotrienes can be separated into the fairly well characterized cysteinyl leukotrienes and less well characterized leukotriene B. Effects of the leukotrienes are mediated through receptors that are expressed on a variety of cell types and can be modulated based on the inflammatory environment present [39]. Human 5-lipoxygenase (5-LOX) is one of the key anti-inflammatory drug targets due to its key role in leukotrienes biosynthesis which show that leukotrienes [40] and low-grade inflammation might be a common denominator of some psychiatric diseases as major and postpartum depression, schizophrenia, and autism [41]. The long-chain polyunsaturated fatty acids (LCPUFA) ecosapentanoic (EPA), docosahexaenoic acid (DHA) and AA are intimately related to the initiation and resolution of inflammatory responses proved in the present study. El-Ansary et al. [33] proved that the balance between AA and EPA + DHA is disturbed in Saudi autistics. Higher DHA/AA, EPA/AA and EPA/DHA in plasma of Saudi autistics could be easily related to the recorded elevation of PGE2 and leukotrienes (Table 1), as lipid mediators involved in the pathophysiology of autism. The significant elevation of PGE2 in autistic patients compared to age-matched control participants could be related to glutamate exitotoxicity [42], lower GABA [8] and autistic amygdala lesion as factor related to the social abnormalities in autism [43]. Lozinsky [44] proved that PGE2 alter intracellular Ca^2+^ homeostasis, glutamate release and activation of transcription factors. PGE2 increase the release of intracellular Ca^2+^ stores, with some entry of extracellular calcium. This in turn could induce glutamate release from astrocytes leading to abnormal neuron-astrocyte interaction. Since elevated glutamate is coupled with decreased GABA due to decreased glutamic acid decarboxylase (GAD) protein expression in partial and cerebellar cortex of brain of autistic individuals, so elevated PGE2 reported in the present study could be related to the reduced GABA level previously recorded in Saudi autistic patients compared to control [8].

Elevated prostaglandins could be related to the recent record of Rossignol and Frye [9] that immune dysregulation/ inflammation are the first etiological factor of autism. It is well known that autism could be associated with viral infections [45], which cause an up regulation in prostaglandins that then mediate fever production. This suggestion could support the elevated inflammatory cytokines in the plasma [7,46] and brains of autistic patients [47,48].

The profound effect of gonadal steroids on the developing brain to produce sex differences in physiology and behaviour suggests steroids may also be an important source of sex differences in neuropathology. While the causes of autism are unknown, several environmental factors are suspected. The “extreme male brain” theory of autism states that excessive prenatal androgens push brain development beyond the range of the normal male and causes a pathological level of hypersensitivity, hyposociality, extreme focus and disordered thoughts [49,50]. It has been shown that children with autism have significantly elevated androgen levels and that anti-androgen therapy may be of benefit in some autistic patients [51-53]. Testosterone is the immediate precursor of estradiol, which is formed via aromatization. The connection between prostaglandins and estradiol regulation which was uncovered by Dean [54] indicate the role of both in the common pathway of cerebellar development and that disruption of each could be related to the risk of manifesting autism later in life. Higher PGE2 reported in the present study could lead and reflect disruption of estradiol level through activation of aromatase enzyme, which could explain the sex differences in autism prevalence.

The significant positive correlations recorded between 8-isoprostan, Leukotriene and PGE2 (Table 2 and Figure 5), confirmed the association between the impairment of LCPUFA metabolism together with pro-inflammation and oxidative stress in the etiopathology of autism. The high specificity and sensitivity recorded for the three measured parameters could help to list 8-isoprostan, Leukotriene and PGE2 among predictive biomarkers usually used to predict outcome to a specific treatment. This study could help to suggest omega-3 LCPUFA supplementation as a strategy for the early intervention to ameliorate pro-inflammation and oxidative stress related symptoms in autism. However, further research is required before definitive recommendations can be made about the routine use of omega-3 fatty acids in treating autistic patients [55].

## Competing interests

The author(s) declare that they have no competing interests.

## Authors’ contributions

El-A: Designed the work, performed the analysis and drafted the manuscript. Al-A: Diagnosis of the autistic patients and provision of blood samples of all participants. All authors read and approved the final manuscript.
